# Using machine learning to estimate the incidence rate of intimate partner violence

**DOI:** 10.1038/s41598-023-31846-8

**Published:** 2023-04-04

**Authors:** Zhuo Chen, Wen Ma, Ying Li, Wei Guo, Senhu Wang, Wansu Zhang, Yunsong Chen

**Affiliations:** 1 School of Social and Behavioral Sciences, Nanjing University, Nanjing, 210023 China; 2 Department of Sociology and Anthropology, National University of Singapore, Singapore, 117573 Singapore; 3 School of Law, Nanjing University, Nanjing, 210023 China

**Keywords:** Human behaviour, Health care

## Abstract

It is difficult to accurately estimate the incidence rate of intimate partner violence (IPV) using traditional social survey methods because IPV victims are often reluctant to disclose their experiences, leading to an underestimation of the incidence rate. To address this issue, we applied machine learning algorithms to predict the incidence rate of IPV in China based on data from the Third Wave Survey on the Social Status of Women in China (TWSSSCW 2010). Specifically, we examined five unbalanced sample-processing methods and six machine learning algorithms, choosing the random under-sampling ensemble method and the random forest algorithm to impute the missing data. Analysis of the complete data showed that the incidence rates of physical violence, verbal violence, and cold violence were 7.10%, 13.74%, and 21.35%, respectively, which were higher than the incidence rates in the original dataset (4.05%, 11.21%, and 17.95%, respectively). The robustness of our findings was further confirmed by analysis using different training sets. Overall, this study demonstrates that better tools need to be developed to accurately estimate the incidence rates of IPV. It also serves as a useful guide for future research that imputes missing data using machine learning.

## Introduction

Domestic violence refers to violence or other abusive behavior that occurs in a domestic environment, including violence against partners, children, adolescents, and the elderly. Recently, intimate partner violence (IPV), a specific type of domestic abuse, has received growing attention in global scholarship^1,2^. IPV is typically defined as any conduct that harms another person physically, psychologically, or sexually while they are involved in an intimate relationship—usually a marriage^3,4^. The CDC (Centers for Disease Control and Prevention)’s report reflects that, in the US, the proportion of women suffering from all forms of IPV in 2016/2017 was 47.3%, and the proportion of men was not far behind at 44.2%^5^. According to the World Health Organization, in 2018, approximately 30% of women worldwide had experienced physical and/or sexual violence by an intimate partner or husband in the previous 12 months^6^. Studies in China using local samples estimated that the incidence rate of IPV was between 20 to 25%^7^, but the national incidence rate of IPV remains unknown due to the absence of reliable nationwide survey data.

Studies have considered IPV as a sensitive issue and suggested a degree of underreporting in the survey data. Specific findings report that women in the US underreport IPV by roughly 50%, while men are more likely than women to underreport it^8^. Regarding specific explanations for underreporting, victims may feel ashamed of being involved in violence or fear that associating with violent partners will humiliate them^9^. This sense of shame deprives victims of their dignity^10^, causing self-blame, self-deprecation, and a self-imposed sense of isolation^11^, which makes them more likely to remain silent^12^. Underreporting due to survey methodology should also be considered, as the context in which the survey is conducted and the characteristics of the surveyor can have a significant impact on the likelihood of a response. Research also shows that women are more likely to downplay their IPV experiences when the interviewer is a man, when other people are around, or when they feel their privacy is threatened^13,14^.

In particular, the emphasis on harmony and tight family ties in traditional Asian values may deter Asian women from reporting IPV, resulting in higher rates of response avoidance^15^. Chinese women are more likely to underreport their partner’s violence against them because the society may stigmatize victims more than perpetrators^16^. Since China has long owned the “face” culture, where self-esteem, social expectations, and relationships are important^17^, Chinese people tends to act to avoid humiliation, especially when it comes to immoral behavior such as IPV. Due to these factors, it is challenging for researchers and policymakers to estimate the true prevalence of IPV, and discussions of the mechanisms based on IPV statistics may also be questioned.

Due to response avoidance, surveys on IPV may have a proportion of non-response (missing values), making the scientific prediction of the proportion of non-response to IPV becomes a critical issue in estimating the incidence of IPV and in the subsequent analysis of its potential effects. Is there a method to accurately predict the answers of non-respondents on this socially sensitive indicator? The assumption of complete random missingness for IPV is not valid for the psychology of some respondents who deliberately conceal the existence of IPV, which means that traditional methods to handling missing values, such as deleting missing value cases and mean interpolation, would be inaccurate. Though more complex interpolation methods, such as regression interpolation, maximum likelihood estimation, and multiple interpolation, can predict IPV based on more information, they are statistical models of parameter estimation and are not good at prediction because of their limitations on the number of variables, the distribution of variables, and the patterns of relationships (e.g., linear relationships)^18^. In this instance, complex machine learning methods with greater computational power offer a novel way of predicting IPV. They can overcome the limitations of model form and variable selection, capture non-linear relationships, and fully account for various interactions between variables. Researchers have demonstrated the excellent performance of machine learning in predicting and imputing missing values^19–21^. To obtain a more accurate estimate of the prevalence of IPV in China, we adopted six machine learning algorithms to analyze data from the Third Wave Survey on the Social Status of Women in China (TWSSSCW 2010) to estimate the incidence rates of three types of IPV: physical violence, verbal violence, and cold violence. This study can remind IPV researchers to be more attentive to the missing proportion in the survey before empirical analysis, and provide a methodological reference for not only IPV prediction but also other sensitive survey questions.

## Method

### Survey and sample

The data were obtained from the TWSSSCW 2010, the most recent nationally representative survey. This survey was conducted and approved by the All-China Women’s Federation and the National Bureau of Statistics of China. All the data are anonymous and were collected following relevant guidelines and regulations. It included a wide range of questions on family, gender relations, health and well-being, and domestic violence (including IPV). The TWSSSCW 2010 used a stratified probability-proportional-to-size multi-stage random sampling design to ensure that the sample was representative of the general population at the provincial/municipal, street, and neighborhood levels^22^. The survey included a sample of 26,166 respondents, but due to the research question of our study, we restricted our sample to women and men who were married. Thus, the sample we used for our analysis involved 23,597 married women and men. The Ethics Committee of the School of Social Sciences of Nanjing University approved this study. Informed consent was obtained from all subjects.

### Measures

Three types of IPV were used as the dependent variables, namely physical violence, verbal violence, and cold violence. In the TWSSSCW 2010, respondents were asked “In your whole married life, has your partner ever hit you?”, “In your whole married life, has your partner ever insulted you?”, and “In your whole married life, has your partner ever constantly ignored you?” and gave responses on a 4-point scale (“never,” “occasionally,” “sometimes,” or “often”). We dichotomized the three variables to measure whether respondents had suffered from IPV. As the frequencies expressed as “occasionally,” “sometimes,” or “often” are similar and vague in meaning, we collapsed these frequencies into one category. Table 1 shows that 1.22% of men and 2.83% of women experienced physical violence; 4.88% of men and 6.32% of women experienced verbal violence and 8.46% of men and 9.49% of women experienced cold violence. The total percentage of missing data was 6.95%. Figure S1 in the supplementary material shows the demographic distribution of missing data on respondents’ experiences of IPV. Of the total missing values for IPV, approximately 65% were female, 60% were urban, and 70% were less educated. Compared to men (35%), rural (40%), and higher educated respondents (30%), the rates of refusal to answer questions about IPV are significantly higher among female, urban, and lower educated respondents.

**Table 1:** Descriptive statistics of IPV and missing cases.

	All sample	Men	Women
Physical violence (%)			
No	95.95	46.57	49.38
Yes	4.05	1.22	2.83
Verbal violence (%)			
No	88.79	42.90	45.90
Yes	11.21	4.88	6.32
Cold violence (%)			
No	82.05	39.34	42.71
Yes	17.95	8.46	9.49
Proportion of missing cases (%)	6.95	2.41	4.54
Observations (n)	23,597	11,063	12,534

To predict IPV missing values, we adopted 611 variables from the questionnaire as features that would be provided as input to the machine learning algorithm used to train the model. These feature variables cover a wide range of topics related to aspects such as respondents’ health, educational level, employment status, family background, gender ideology, leisure activities, political participation, religious affiliation, housework hours, and insurance enrolment status, providing rich clues for IPV prediction.

### Imbalanced data problem

Table 1 demonstrates how unbalanced the dataset was because only a small percentage of respondents reported having experienced IPV. Since most classifiers work with data drawn from the same distribution as the training set, using a standard prediction algorithm may lead to an underprediction of the percentage of respondents who had experienced IPV. Thus, it would be challenging to prepare appropriate data for training and testing, resulting in incorrect predictions. For example, if 99% of respondents reported that they had not experienced IPV, a standard machine learning algorithm, such as a naïve Bayesian classifier or a decision tree, would struggle to make accurate predictions for the minority group due to low variation. The two main types of approaches applied to address this problem are over-sampling the minority group and under-sampling the majority group, while each approach has its strengths and limitations. We compared five different resampling methods, including two over-sampling methods (the random over-sampling method^23^ and the synthetic minority over-sampling technique, SMOTE^24^), two under-sampling methods (the random under-sampling ensemble method^25^ and the *K*-means method^26^), and the SMOTE-edited nearest neighbor method^27^ (SMOTE-ENN, which combines over- and under-sampling techniques). We divided the 21,956 non-missing cases into a training set (70%) and a validation set (30%), which had the same distribution of IPV cases as the original dataset. Next, we re-sampled the training dataset, used the random forest method to train the classifier, and finally tested the classifier on the validation set. After comparing the results of the different re-sampling methods, we found that the random under-sampling ensemble method gave the best performance in terms of the accuracy rate, the recall rate, the receiver operating characteristic (ROC) curve, and detection error tradeoff (DET) curve (as detailed in Table S1 and Figure S2 in the supplementary material).

### Algorithm and parameter selection

We compared six different machine learning algorithms: random forest algorithm^28^, adaptive boost algorithm^29^, Gaussian naïve Bayes algorithm^30^, support-vector machine^31^ (SVM), logistic regression algorithm^32^, neural network^33^, and one traditional interpolation method: multiple imputation^34^. Table S2-S4 in the supplementary material lists the accuracy and recall rates in the training and test sets for these algorithms. Generally, accuracy is crucial. However, when predicting IPV, given that the number of victims of IPV is quite small compared to the number of non-victims, it is unreasonable to blindly assume that all 21,956 of the sample would be classified as Category 0 who have never been hit by their spouse and easily achieve 96% accuracy. In this context, recall (the percentage of victims who are correctly predicted) is of great significance. The random forest algorithm can balance the prediction accuracy and recall for “physical violence” with 0.71 and 0.75, respectively.

The random forest algorithm is a supervised learning algorithm that performs classification by constructing multiple decision trees based on training datasets and predicts classification or average scores of individual decision trees (more details on the random forest algorithm are given in the supplementary material). We used the grid search method to search the hyperparameter space for the best cross-validation score, specifying the maximum feature range as 20 to 45 and the maximum depth range as 5 to 30 (see Figure S4 in the supplementary material). We then adopted the random forest algorithm to train the models 500 times, and the majority voting method to determine the final classification. To evaluate the models, we first used tenfold cross-validation to divide the resampled data into 10 consecutive folds and then applied one fold as the test set and the remaining nine folds as the training set. The training result was evaluated in terms of the area under the curve and the ROC and DET curves.

### Robustness and heterogeneity analyses

To ensure the robustness of our findings, we examined whether using different training sets would lead to similar results. Respondents’ propensity to report IPV is associated with their gender ideology. A more patriarchal ideology holds that men are more capable than women and therefore men should predominate in roles of leadership and authority^35–37^. Consequently, men and women who hold a patriarchal gender ideology are more likely to conceal having experienced IPV: men due to fear of stigma^38^, while women due to their desire to protect their partner^39,40^. We adopted the following question to measure patriarchal gender ideology: “Do you think that women are less capable than men?” We assumed that respondents who answered in the affirmative were more likely to have a patriarchal gender ideology; therefore, they tended to provide unreliable answers to the IPV questions. By excluding this particular group of respondents, we were able to replicate our analysis with the random forest algorithm.

## Results

Table 2 reveals that the random forest classifier analyzed 1,641 missing cases of physical violence, of which 854 and 787 were classified as “No” and “Yes,” respectively. Thus, the incidence rate of physical violence was 7.10% in the data imputed by the random forest algorithm, higher than the rate (4.05%) in the original raw dataset (i.e., without imputation). Similarly, the random forest classifier analyzed 1,643 missing cases of verbal violence and classified 860 and 783 cases as “No” and “Yes,” respectively. This equated to an incidence rate of verbal violence of 13.47%, higher than the original incidence rate of 11.21%. Finally, the random forest classifier analyzed 1,645 missing cases of cold violence and classified 546 and 1,099 cases as “No” and “Yes,” respectively. This equated to an incidence rate of cold violence of 21.35%, which was greater than the original incidence rate of 17.95%. Reassuringly, these analyses produced similar results in robustness analyses using different training data (see Table S5 in the supplementary material).

**Table 2:** Fitness statistics and predicted outcomes of the random forest algorithm.

	Physical violence	Verbal violence	Cold violence
T-Acc	0.75	0.77	0.86
V-Acc	0.71	0.68	0.63
T-Rec	1	1	1
V-Rec	0.75	0.72	0.68
AUC	0.81	0.78	0.72
Predicted frequencies			
No	854	860	546
Yes	787	783	1099
Proportions in non-missing data	4.05%	11.21%	17.95%
Proportions in predicted missing data	47.96%	47.65%	66.81%
Proportions in full data	7.10%	13.74%	21.35%

*T-Acc* accuracy in training set; *V-Acc* accuracy in validation set; *T-Rec* recall in training set; *V-Rec* recall rate in test set; *AUC* area under the ROC curve.

**Figure 1:**
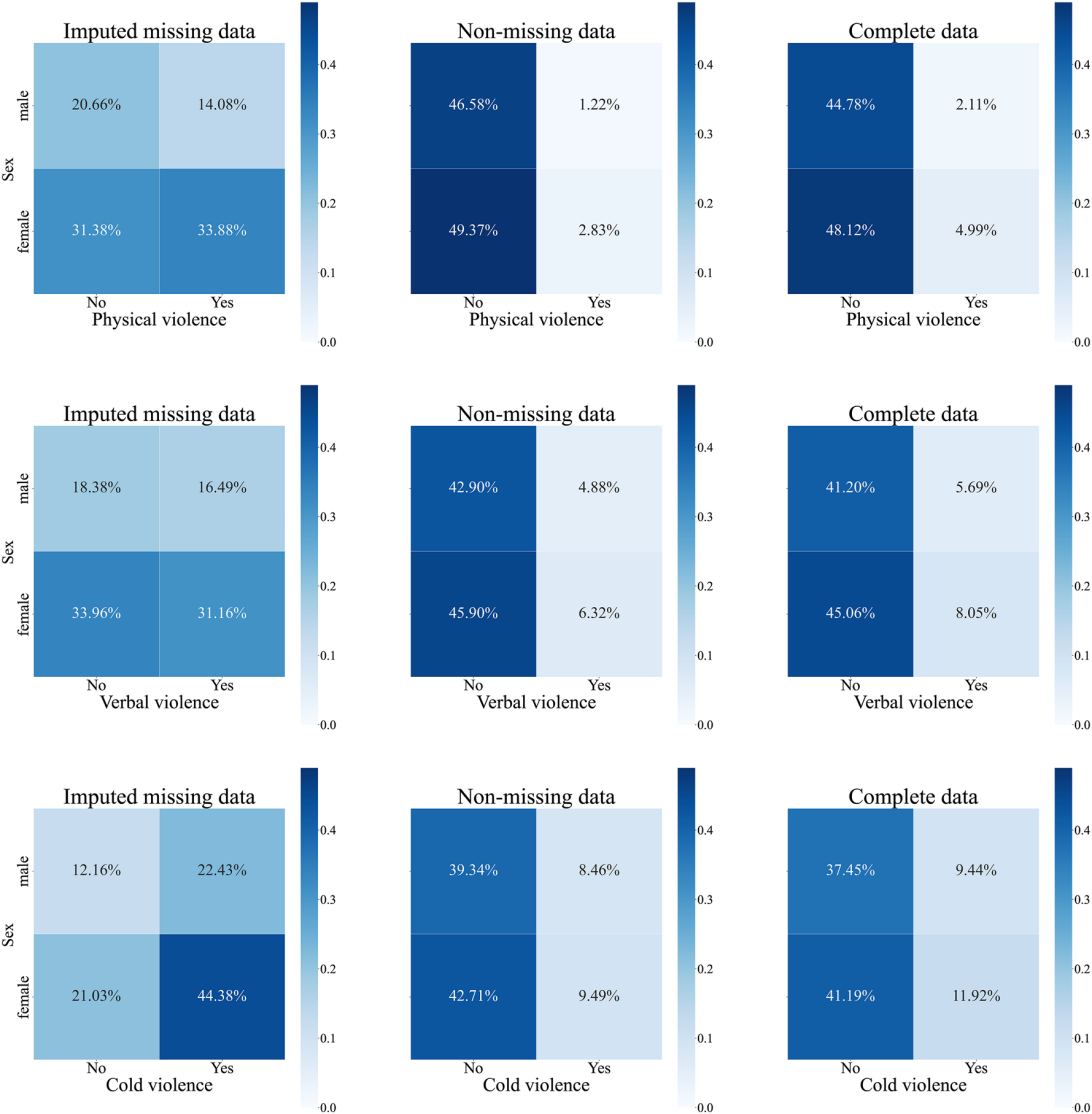
Joint probability distributions of sex and IPV.

Figure 1 shows the joint distributions of IPV in different sex groups, which supports the random forest algorithm’s inference of the missing data that there were much higher incidence rates of IPV than previously reported, especially among women. This implies that the incidence rates of IPV may be underestimated if missing data are not taken into account. When considering the dataset that included imputed missing data, the incidence rate of physical violence increased from 2.83% to 4.99% for women and from 1.22% to 2.11% for men; the incidence rate of verbal violence increased from 6.32% to 8.05% for women and from 4.88% to 5.69% for men; and the incidence rate of cold violence increased from 9.49% to 11.92% for women and from 8.46% to 9.43% for men.

**Figure 2:**
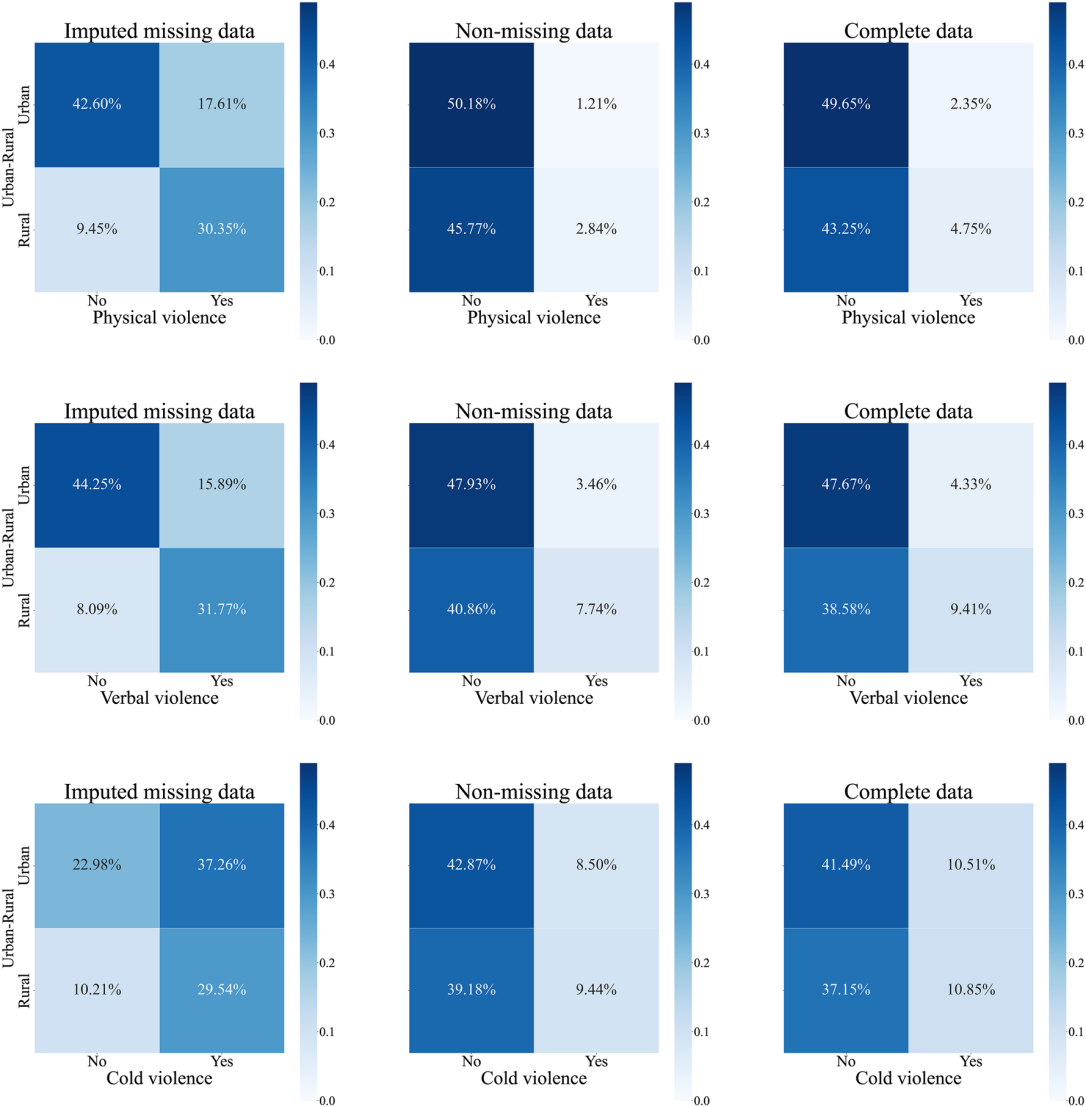
Joint probability distributions of residence and IPV.

**Figure 3:**
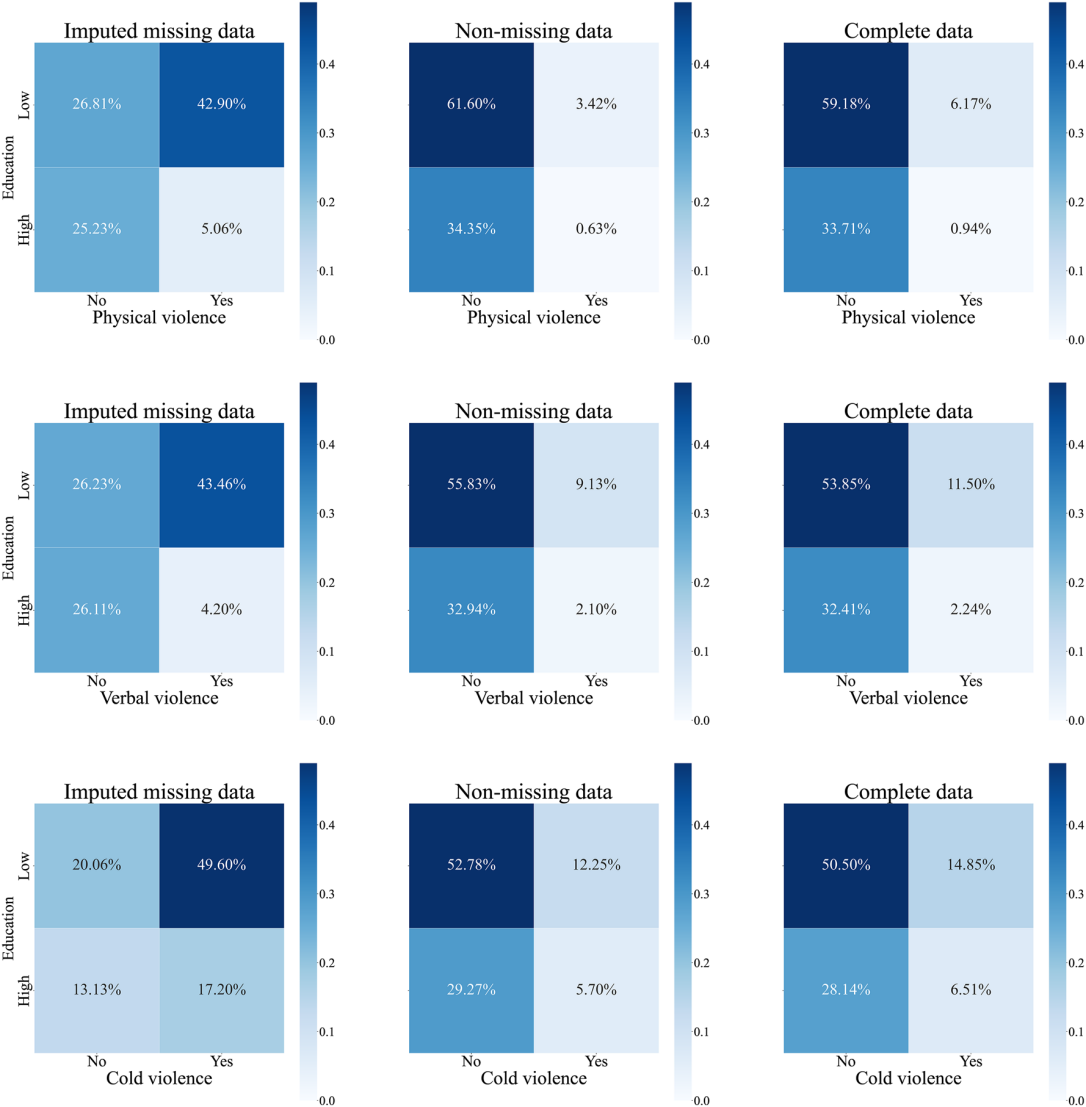
Joint probability distributions of education and IPV. *Note Low* Junior high school or below, *High* High school or above.

Similarly, Fig. 2 illustrates the joint distributions of IPV in both rural and urban areas. According to the imputed missing data, more rural cases (30.35% and 31.77%, respectively) than urban cases (17.61% and 15.89%, respectively) were expected to have experienced physical and verbal violence. Conversely, the urban sample was predicted to have a larger percentage of people encountering cold violence (37.26% in urban and 29.54% in rural areas). When adding the imputed data to the database, the prevalence of physical violence increased from 1.21% to 2.35% for urban residents and from 2.84% to 4.75% for rural residents, the prevalence of verbal violence increased from 3.46% to 4.33% for urban residents and from 7.74% to 9.41% for rural residents, and the prevalence of cold violence increased from 8.5% to 10.51% for urban residents and from 9.44% to 10.85% for rural residents. We also constructed joint probability distributions of IPV in different education groups (see Fig. 3) and found that respondents with lower levels of education consistently had higher incidence rates of the three types of IPV, both before and after imputing missing data.

## Conclusions

So far, the problem of domestic violence, particularly IPV, has received increasing attention from academics and policymakers worldwide, as it is considered to be an important predictor of couples’ health, well-being, and quality of life. However, missing data on certain types of domestic violence poses a substantial challenge to researchers conducting IPV studies. The pattern of these missing data is missing not at random because these missing data are largely related to IPV. For instance, people may not report having experienced IPV because of privacy concerns, fear of reprisal or stigma, or a desire to protect the perpetrator. Traditional approaches used to deal with datasets with missing data, such as listwise deletion and multiple imputation, are not suitable for handling with datasets with missing IPV data: simple interpolation is not appropriate for sensitive indicators such as IPV because it violates the assumption of missing completely at random; traditional model interpolation such as multiple interpolation does not perform well in prediction because of its limitations on the number of variables, the distribution of variables, and pre-defined patterns of data relationships. The aforementioned methods are also ineffective for interpolating data with a high degree of imbalance. Indeed, we found that the incidence rates of physical, verbal, and cold violence were underestimated when we performed listwise deletion or multiple interpolation with missing values. Thus, we applied novel machine learning methods to predict the missing data on the incidence rates of three types of IPV: physical violence, verbal violence, and cold violence.

Therefore, we compared the fitness statistics of different machine learning methods and selected the optimal method—the random forest algorithm—to impute missing data. We discovered that analysis of datasets with imputed data resulted in increased incidence rates of physical violence (7.10%), verbal violence (13.74%), and cold violence (21.35%), compared with analysis of datasets without imputed data (4.05%, 11.21%, and 17.95%, respectively). These results reveal the incidence rate of IPV in China, reduce measurement bias due to intentional concealment by respondents, and provide data and methodological support for correcting this sensitive social survey indicator. We suggest that when IPV researchers work with IPV data with high missing values, they should first predict these indicators before conducting further analyses. At the same time, we hope that our efforts in the analysis method will provide a reference for interpolating missing values and predicting social indicators in the future. In addition to the need for social governance, our results will help government policymakers to understand the true incidence and predict highly sensitive and subjective hidden indicators such as drug use, sexual orientation^41^, AIDS^42^, surrogacy, extramarital affairs, and crime, and thus better prevent and address the social problems related to marginal groups.

However, there are some limitations to this study. First, the data we used was from 2010. We hope that subsequent studies will be able to use more recent data. Second, for machine learning, the original structure and quality of the data determine the upper limit of accuracy. Although we try to minimize the error, any algorithm or resampling effort is less precise than collecting a larger number of minority class samples. Third, predicting whether an individual had experienced IPV is a probabilistic prediction. Our prediction aims to be as close to reality as possible but does not represent the actual state of the sample. Fourth, the prevalence of IPV predicted in this paper is based on married groups only. Although unmarried or extramarital IPV is common, we did not analyze it due to the limitations of the secondary data. Fifth, we noted that several recent studies focused more on the overreporting issue of IPV in Western societies^43,44^. Given the various sociocultural environments in different regions, we suggest that this study’s analytical tools and findings based on the underreporting issue of IPV should be carefully considered before applying in other regions.

## Supplementary Information

Supplementary Information.

## Data Availability

Given that the data we applied in the study, the Third Wave Survey on the Social Status of Women in China (2010) is second-hand, and the unauthorized disclosure of a third party is not allowed under the data processing agreement, we are afraid that we are not able to show these data in the link. Basic information about the data can be found at http://www.china.com.cn/zhibo/zhuanti/ch-xinwen/2011-10/21/content_23687810.htm. Readers can request the data from the China Women’s Federation.

## Code availability

Code related to this paper are available on GitHub (https://github.com/UdvIPV/Unspeakable-domestic-violence).
