# Supplementary material for: Using machine learning to estimate the incidence rate of intimate partner violence
Source: Sci Rep. 2023 Apr 4;13:5533. doi: 10.1038/s41598-023-31846-8 (PMC10073277; doi:10.1038/s41598-023-31846-8)
Supplement: Supplementary file 1 — Supplementary Information. [file 41598_2023_31846_MOESM1_ESM.pdf]

Using machine learning to estimate the incidence rate of intimate partner violence

Zhuo Chen[1], Wen Ma[1], Ying Li[1], Wei Guo[1], Senhu Wang[2*], Wansu Zhang[3*], Yunsong Chen[1*]

[1] School of Social and Behavioral Sciences, Nanjing University, Nanjing, 210023, China

[2] Department of Sociology and Anthropology, National University of Singapore, 117573, Singapore

[3] School of Law, Nanjing University, Nanjing, 210023, China

*Correspondence and requests for materials should be addressed to Y.C.(email: yunsong.chen@nju.edu.cn) or S.W.(email: socsw@nus.edu.sg) or W.Z.(email: zhangwansu@nju.edu.cn)

Supplementary material

Introduction to the random forest algorithm

The random forest algorithm is a supervised learning algorithm used to perform classification. It generates an ensemble of trees, and these then vote for the most popular class. The growth of each tree in the ensemble is governed by random vectors. First, a random vector Θk is generated for the kth tree; it is independent of the previous random vectors $\Theta 1, \ldots, \Theta k-1$, but has the same distribution. Then, a tree is grown using the training set and Θk, which results in a classifier $h(x, \Theta k)$. After a large number of trees have been generated, these trees vote for the most popular class. Thus, a random forest algorithm is a classifier that consists of a set of tree-structured classifiers $\{h(x, \Theta k), k = 1, \ldots\}$, where $\{\Theta k\}$ are independent identically distributed random vectors and each tree casts a unit vote for the most popular class at input x (Breiman, 2001). This can be expressed by the following formula:

$$H(x) = \arg\ max \sum_{i=1}^{k} I(h_i(x) = Y)$$

where *H(x)* represents the random forest model, $h_i(x)$ is a single tree-structured classifier, and Y is the output variable or target variable.

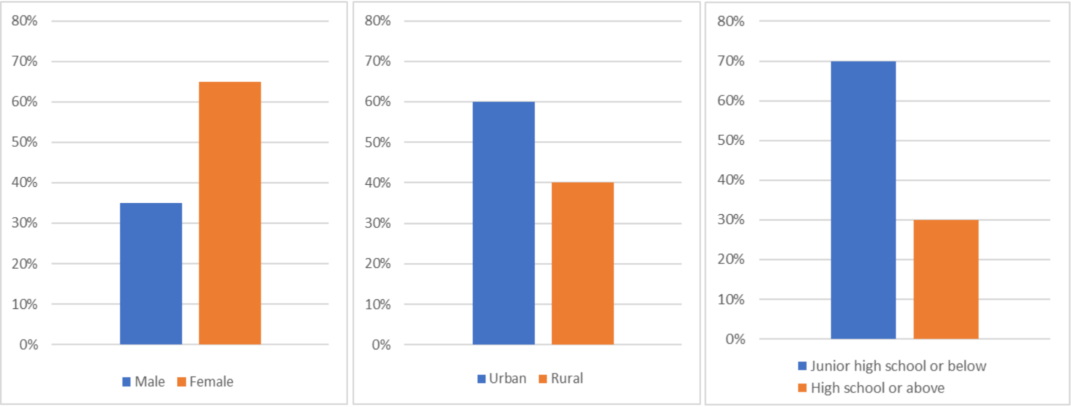

Figure S1. Social and demographic distributions of cases with missing IPV data

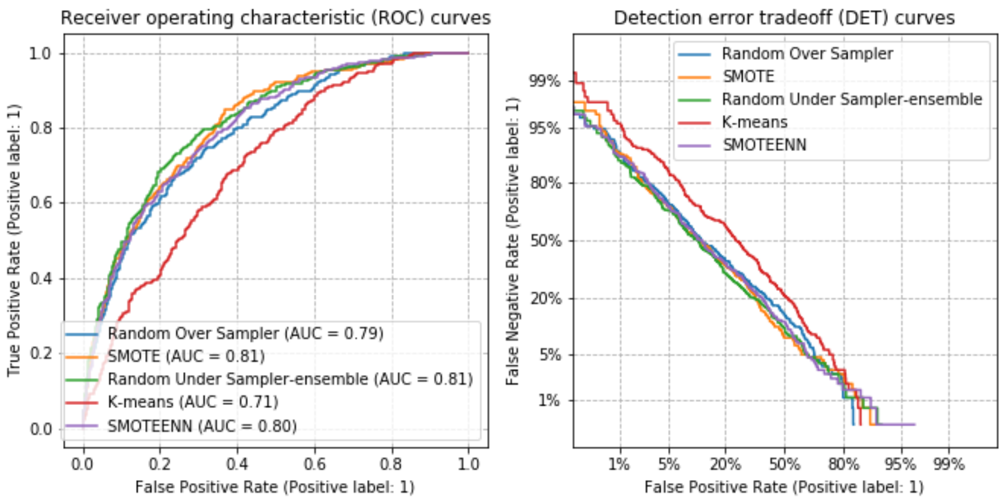

Figure S2. ROC and DET curves for different sampling methods for the dependent variable "physical violence."

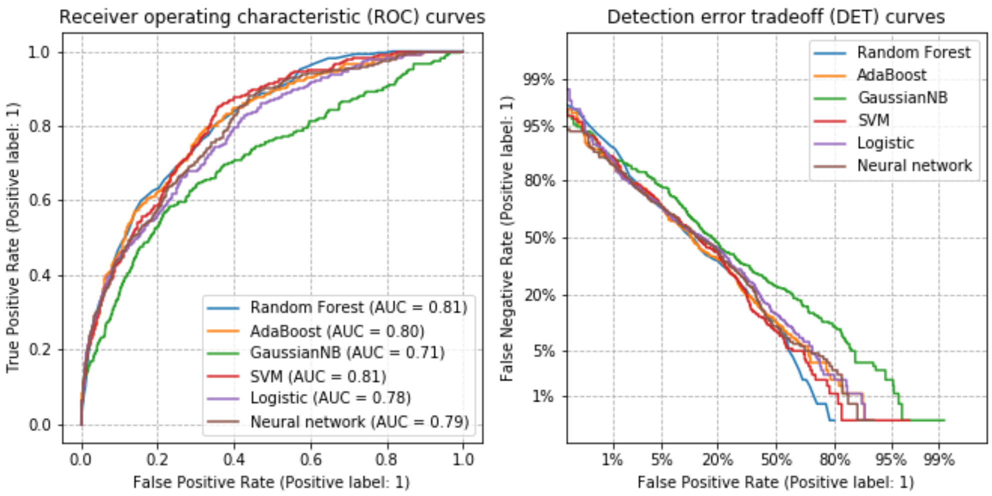

Figure S3. ROC and DET curves for different algorithms for the dependent variable "physical violence."

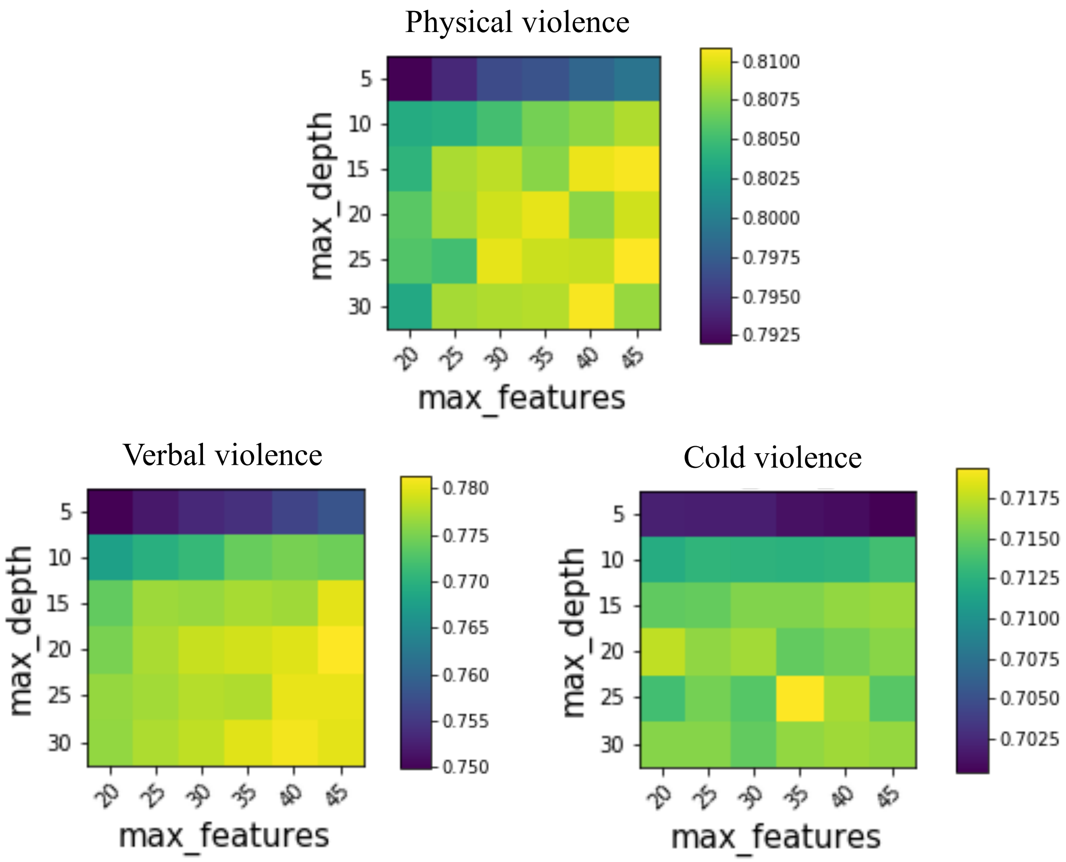

Figure S4. Area under the curve (AUC) metrics for different parameter settings of the random forest algorithm.

Table S1. Fitness statistics for the dependent variable "physical violence" in various sampling methods.

	T- Acc	V- Acc	T- Rec	V-Rec	TN	FN	FP	TP
ROS	0.88	0.85	0.95	0.53	5457	863	126	141
SMOTE	0.96	0.95	0.33	0.16	6229	91	224	43
RUS-ensemble	0.72	0.71	0.95	0.77	4499	1821	62	205
K-means	0.28	0.27	1.00	0.96	1543	4777	11	256
SMOTE-ENN	0.95	0.94	0.47	0.25	6096	224	200	67

Note. T-Acc = accuracy in training set, V-Acc = accuracy in test set, T-Rec = recall in training set, V-Rec = recall in test set. TN = true negative, FN = false negative, FP = false positive, TP = true positive, ROS=random oversampling method, SMOTE= synthetic minority oversampling technique, RUS-ensemble= random under-sampling ensemble method, SMOTE-ENN= SMOTE-edited nearest neighbor method.

Table S2. Fitness statistics for the dependent variable "physical violence" in various algorithms.

	T- Acc	V- Acc	T- Rec	V-Rec	AUC	Proportions
Random Forest	0.75	0.71	1	0.75	0.81	7.10%
AdaBoost	0.80	0.79	0.81	0.63	0.80	5.38%
Gaussian NB	0.58	0.58	0.86	0.72	0.71	7.00%
SVM	0.82	0.80	0.88	0.58	0.81	7.73%
Logistic	0.79	0.77	0.86	0.59	0.78	6.45%
Neural network	0.82	0.78	1.00	0.58	0.79	10.59%
Multiple Imputation	-	0.93	-	0.12	0.54	4.18%

Note. T-Acc = accuracy in training set, V-Acc = accuracy in validation set, T-Rec = recall in training set, V-Rec = recall in validation set, AUC=Area Under ROC Curve, Proportions= physical violence rate in full data.

TableS3. Fitness statistics for the dependent variable “verbal violence” in various algorithms.

	T- Acc	V- Acc	T- Rec	V-Rec	AUC	Proportions
Random Forest	0.77	0.68	1.00	0.72	0.78	13.74%
AdaBoost	0.74	0.73	0.76	0.68	0.78	11.99%
Gaussian NB	0.43	0.41	0.89	0.82	0.69	10.97%
SVM	0.82	0.76	0.89	0.60	0.78	10.73%
Logistic	0.75	0.73	0.79	0.65	0.77	12.44%
Neural network	0.84	0.76	1.00	0.62	0.78	12.57%
Multiple Imputation	-	0.82	-	0.21	0.55	11.26%

Note. T-Acc = accuracy in training set, V-Acc = accuracy in validation set, T-Rec = recall in training set, V-Rec = recall in validation set, AUC=Area Under ROC Curve, Proportions= verbal violence rate in full data.

TableS4. Fitness statistics for the dependent variable “cold violence” in various algorithms.

	T- Acc	V- Acc	T- Rec	V-Rec	AUC	Proportions
Random Forest	0.86	0.63	1.00	0.68	0.72	21.35%
AdaBoost	0.69	0.68	0.66	0.65	0.72	20.53%
Gaussian NB	0.42	0.40	0.86	0.81	0.62	21.69%
SVM	0.80	0.70	0.86	0.58	0.72	17.12%
Logistic	0.69	0.67	0.69	0.62	0.71	18.76%
Neural network	0.85	0.71	1.00	0.57	0.72	18.86%
Multiple Imputation	-	0.73	-	0.25	0.54	17.99%

Note. T-Acc = accuracy in training set, V-Acc = accuracy in validation set, T-Rec = recall in training set, V-Rec = recall in validation set, AUC=Area Under ROC Curve, Proportions= cold violence rate in full data.

Table S5. Results from the random forest algorithm processing of different training sets.

Training data	Training sample	Physical violence			Verbal violence			Cold violence		
		Men	Women	Total	Men	Women	Total	Men	Women	Total
Original incomplete data	——	1.22%	2.83%	4.05%	4.88%	6.32%	11.21%	8.46%	9.49%	17.95%
Complete data with imputations	21,956	2.11%	4.99%	7.10%	5.69%	8.05%	13.74%	9.43%	11.92%	21.35%
Complete data, excluding respondents with patriarchal gender ideology	18,290	2.23%	5.31%	7.54%	5.80%	8.38%	14.18%	9.31%	11.82%	21.13%
